# Nutritional-Related Predictors of Anemia among Pregnant Women Attending Antenatal Care in Central Ethiopia: An Unmatched Case-Control Study

**DOI:** 10.1155/2020/8824291

**Published:** 2020-11-19

**Authors:** Berhanu Senbeta Deriba, Gizachew Abdissa Bulto, Elias Teferi Bala

**Affiliations:** ^1^Salale University, Department of Public Health, Fiche, Ethiopia; ^2^Ambo University, Department of Midwifery, Ambo, Ethiopia; ^3^Ambo University, Department of Public Health, Ambo, Ethiopia

## Abstract

**Background:**

Anemia is a major public health problem in both developed and developing countries especially among pregnant women. Nearly half of pregnant women in Ethiopia have anemia which has both health and economic impacts. Therefore, this study is aimed at identifying nutritional-related predictors of anemia among pregnant women attending antenatal care in Central Ethiopia, 2019.

**Methods:**

An unmatched case-control study was conducted at public hospitals in Central Ethiopia from February to April 2019. The consecutive sampling technique was used to select study participants. Data were collected by a structured questionnaire, and the collected data were entered into Epi Info version 7 and SPSS version 23 for analysis. Binary and multiple logistic regression analyses were computed to identify predictors of anemia. Adjusted odds ratio (AOR) with 95% confidence interval (CI) and *p* value < 0.05 was used to determine the presence of an association.

**Result:**

A total of 426 pregnant women (142 cases and 284 controls) had participated in this study. Taking tea/coffee immediately after food (AOR = 2.35, 95% CI: 1.39-3.99), mid-upper arm circumference (MUAC) of mothers of <23 centimeters (AOR = 3.83, 95% CI: 2.26-6.49), the presence of forbidden food during pregnancy (AOR = 2.21, 95% CI: 1.24-3.88), not taking additional food (AOR = 1.99, 95% CI: 1.17-3.40), unable to take fruit (AOR = 4.05, 95% CI: 1.3-15.47), loss of appetite (AOR = 2.28, 95% CI: 1.28-4.09), low dietary diversity score (DDS) (AOR = 3.29, 95% CI: 1.83-5.90), and medium DDS (AOR = 2.88, 95% CI: 1.46-5.70) were found to be determinants of anemia.

**Conclusions:**

Taking tea or coffee immediately after food, MUAC of mothers, the presence of forbidden food, not taking additional food, frequency of taking fruit, and dietary diversity were predictors of anemia among pregnant women. Therefore, interventions targeted at prevention of anemia among pregnant mothers should emphatically consider those identified determinants. This finding also highlights the need for strong nutritional counseling to prevent anemia among pregnant mothers during antenatal care follow-ups along with other interventions.

## 1. Introduction

Anemia in pregnancy is defined as a hemoglobin level below 11 gm/dl [1]. It is a worldwide public health problem disturbing both developing and developed countries with the consequences of health impact and economic development [1]. The causes of anemia can be nutritional and nonnutritional. The leading cause of anemia throughout the world is nutritional anemia which is particularly common in women of childbearing age and specifically during pregnancy [2]. Anemia is the most common condition that predominantly affects women of childbearing age especially pregnant mothers [3]. Although anemia occurs at all stages of life, it is highly prevalent among pregnant women because of the need for iron for women themselves and their fetus [1]. Globally, 38% (32.4 million) of pregnant women aged 15-49 years were affected by anemia [4]. The prevalence of anemia among reproductive-age women was 23% in Ethiopia and 26.2% in Oromia Regional State [5].

In low-income countries, iron deficiency anemia in pregnancy attributes about half of all types of anemia to improper nutrition, living in an unhygienic environment, high infection burden, lack of health care facilities and proper utilization, low educational status, early marriage, short birth interval, and lack of awareness of antenatal care [6]. Ethiopia is one of the developing countries in Africa that has a high problem of food insecurity and a high burden of malnutrition which leads to anemia [7].

Anemia during pregnancy becomes an important public health problem in Ethiopia because it results in high rates of maternal mortality, preterm birth, low birth weight, and other health problems. The magnitude of maternal mortality ratio in Ethiopia was high (412 per 100,000) [5]. It is important to identify risk factors that contribute to the development of anemia in pregnant women to successfully prevent it and implement a global nutrition target of reducing the magnitude of anemia among pregnant women by half [8].

Even though the Ethiopian Federal Ministry of Health developed a national nutrition strategy to improve the micronutrient deficiency among pregnant women by increasing the system to give comprehensive and routine nutritional assessment intervention as well as routine iron and folic acid supplementation and deworming during pregnancy, anemia is still a public health problem among pregnant mothers in Ethiopia [7, 9, 10].

Despite the interventions, the magnitude of anemia was still high and results in significant morbidities and mortalities among pregnant women and had high economic losses in Ethiopia. Most of the studies previously conducted only focused on health facilities within one town and on determining the risk factors of anemia. Moreover, majority of the previous studies conducted in Ethiopia were cross-sectional study which is weak in displaying the real association between anemia and its predictors [11–20]. In previous studies, nutritional-related predictors of anemia like food taboo, additional food during pregnancy, and food frequency were not studied. Even though food taboo is very common in Ethiopia, whether it is a predictor of anemia or not was not studied in the previous study. Moreover, most of the previous cross-sectional studies recommended a case-control study (analytic study) [11, 13, 16, 21, 22]. Hence, considering the variation of a sociodemographic and economic characteristic of the study population which may affect the status of anemia among pregnant women is important. Furthermore, knowing the predictors of anemia is important in providing targeted intervention for prevention of anemia and its complications on pregnant mothers and their fetus in Ethiopia. Therefore, unlike the previous studies, the current study tried to identify nutritional-related predictors of anemia among pregnant women attending antenatal care at all public hospitals (by including all hospitals present in urban and rural) in West Shewa Zone, Oromia Regional State, Central Ethiopia, by focusing on nutritional-related predictor of anemia.

## 2. Methods

### 2.1. Study Design and Setting

An institutional-based unmatched case-control study was conducted from February to April 2019 in eight public hospitals in the West Shewa Zone, Oromia Region, Central Ethiopia. Ambo Town is the zone's capital which is located 114 kilometers far from the capital city of the country, Addis Ababa. The West Shewa Zone is one of the 20 zones found in Oromia Regional State. It has a total population of 2,650,781 from which 91,982 were pregnant women according to the zonal health office report of January 2019 [23]. The zone has eight hospitals and 90 health centers. All hospitals give 24 hours of maternal health care services.

### 2.2. Source and Study Population

The source populations were all pregnant women (15-49 years of age) attending the first antenatal care follow-up at public hospitals in West Shewa Zone during the data collection period. The study populations were all pregnant women (15-49 years of age) attending the first antenatal care follow-up at public hospitals in West Shewa Zone during the data collection period, and those with a hemoglobin level of <11 g/dl were considered as cases (anemic women), whereas those with hemoglobin level greater than or equal to 11 g/dl were considered as controls (nonanemic women) according to WHO definition for the diagnosis of anemia in pregnancy [1]. Pregnant women who were severely ill and unable to respond were excluded from the study.

### 2.3. Sample Size Determination and Sampling Technique

Two population proportion formulas were used for sample size calculation and calculated through Epi Info version 7 statistical software package by considering confidence level 95% (*Zα*/2 = 1.96), power 80% (*Zβ* = 0.84), and a case-control ratio 1 : 2. By taking a case-control study conducted in Durame Town, one significantly associated variable was not eating additional food during the current pregnancy, where P1 is the proportion of cases exposed (42.1%) and P2 is the proportion of controls exposed (18.8%) and AOR = 2.5. Considering a 10% nonresponse rate, the final sample size was 435 pregnant women of which 145 of them were cases and 290 were controls.

**Figure 1:**
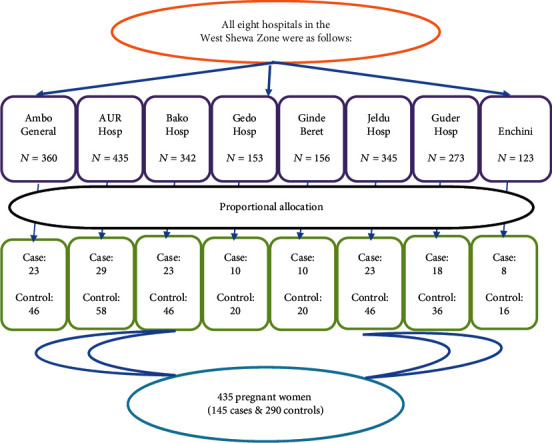
Schematic presentation of pregnant women attending ANC in public hospitals of West Shewa Zone from February to April 2019. *N*: total number of first ANC attendees; AUR: Ambo University Referral Hospital.

The study was conducted in all hospitals available in the zone. The numbers of study participants were allocated to each selected hospital proportional to their average client size attended per month by referring to the registration books of each antenatal one care unit. Therefore, the sample of each hospital was calculated by multiplying the average number of pregnant women attending first antenatal care in each hospital per month from their last quarter performance with total sample size (435), divided by the total number of pregnant women attending the antenatal care unit per month (2187). Finally, the study participants were selected at each hospital using consecutive sampling techniques which means after collecting data from one case, data were also collected from two cases that came after that case (Figure 1).

### 2.4. Operational Definitions

Anemia was defined based on WHO criteria using the hemoglobin level adjusted at sea level altitude and gestational age. So, anemia in pregnancy was defined as when the hemoglobin level is below 11 gm/dl [1]. Those with a hemoglobinlevel < 11 g/dl were considered cases (anemic), whereas those with a hemoglobin level greater than or equal to 11 g/dl were considered controls (nonanemic) according to WHO definition for the diagnosis of anemia in pregnancy [1].

The dietary diversity score (DDS) was calculated from a single 24-hour dietary recall data. All the foods and drinks or liquids used up or consumed a day before the study day were categorized into nine food groups. The nine specified food groups were (1) starchy staple: cereals (rice, wheat), roots, tubers, and plantains; (2) pulses and legumes (lentils, beans, and peas); (3) oilseeds and nuts; (4) milk product; (5) fish, meat, and poultry; (6) eggs; (7) fruits and vegetables rich in vitamin A; (8) green leafy vegetables; and (9) other fruits and other vegetables. Consuming the food item from any of the groups was given score 1 and score 0 if no food was consumed. Thus, a dietary diversity score of 9 points was computed by adding the values of all the groups. Then, after, it was categorized as low if DDS ≤ 3, medium if DDS is between 4 and 6, and high if DDS is 7-9 [24].

Taking tea or coffee immediately after a meal is drinking tea or coffee without staying at least for 1-2 hours after the food is eaten which inhibits iron absorption [25].

### 2.5. Data Collection Tool and Procedure

Data was collected through a face-to-face interview by standardized structured questionnaires; the majority of which were adapted and modified from the previous study [26], and some of the questionnaires were developed from earlier similar articles. The questionnaire was prepared in English and translated to local language for better understanding for both data collectors and respondents and translated back to an English version by language experts to check the consistency. Dietary intake (24-hour recall) questions were adapted and modified from Food and Agriculture Organization (FAO) guidelines [24]. Eight BSc midwife professionals and two senior midwife professionals who have previous experience of supervision were recruited for data collection and supervision, respectively. Information related to dietary habit (nutritional-related factors) of respondents was collected through a face-to-face interview by using a pretested structured questionnaire which was done after laboratory test results were obtained because cases and controls cannot be identified before the hemoglobin test result. The women dietary diversity score (WDDS) was calculated based on the Food and Agricultural Organization (FAO) guidelines. The score was computed and changed into three categories (low, medium, and high) [24]. Mid-upper arm circumference (MUAC) of mothers was measured to the nearest 0.1 cm to determine the nutritional status of the mother by data collectors at the time of data collection using a nonstretchable tape meter.

#### 2.5.1. Specimen Collection and Processing

Specimen collection and processing were conducted at eight hospitals by eight trained laboratory technologists. Two experienced and trained laboratory technologists supervised every step of specimen collection, processing, and analysis. The blood for hemoglobin concentration was taken based on standard operation procedures (SOPs). A venous blood sample of 10 ml was taken from study participants, by using a HemoCue Hb 301 analyzer (manufactured by HemoCue AB); a precalibrated instrument was designed for measurement of hemoglobin concentration and labeled with an identification number. The venous blood was drawn through micro cuvettes and inserted into the HemoCue Hb analyzer, and the result was recorded.

### 2.6. Data Quality Control and Management

The training was given for data collectors and supervisors on the objective of the study, contents of the questionnaire, confidentiality, the right of respondents, and how to collect data. The questionnaire was pretested on 5% of the sample at an unselected hospital to assess the reliability of the data collection instruments and findings. Investigators, data collectors, and supervisors discussed the questionnaire and modified tools for any inconsistencies and ambiguity after the pretest. The laboratory procedure quality was assured by giving training for laboratory technologists through a standard operating procedure (SOP) and regular monitoring of reagents, expiry date, and proper storage. Data collectors and supervisors checked the collected data for consistency and completeness every day at the end of each data collection day, and necessary corrective measures were taken accordingly.

### 2.7. Data Processing and Analysis

Data were checked for completeness and coded, cleaned, and entered to Epi Info version 7 and transported to SPSS version 23 for data cleaning and analysis. Descriptive statistics such as tables, graphs, and proportions were used to present data. Bivariate and multivariate logistic regression analyses were carried out to determine the presence of an association between anemia and independent variables. Variables with a *p* value less than 0.25 at 95% CI in the bivariate logistic regression were selected as a candidate and entered into a multivariate logistic regression [27]. Multiple logistic regressions were carried out to identify predictors of anemia among pregnant women. The model goodness of the test was checked by Hosmer-Lemeshow goodness fit, and the *p* value of the model fitness test was 0.870. Multicollinearity and confounding effect were checked by using standard error which was less than two, and no collinearity exists between the independent variables. Then, all candidate variables were entered into a multivariate model since no collinearity was found between them. Finally, AOR with 95% CI and *p* value < 0.05 was considered statistically significant.

### 2.8. Ethical Consideration

Ethical clearance was obtained from Ambo University, College of Medicine and Health Sciences Institutional Review Committee. Written consent was obtained from study participants where the study participants were ≥18 years of age, and written assent was obtained from the guardian for those <18 years of age after clarifying the objective and aim of the study. The confidentiality and privacy of the study participants were also maintained rigorously. Those mothers who were found to have anemia, malnutrition, and parasites were treated and linked to appropriate units in the hospitals for follow-up.

## 3. Results

### 3.1. Sociodemographic-Related Characteristics

A total of 426 pregnant women (142 cases and 284 controls) had participated in this study. Sixty-five (45.8%) cases and 159 (56%) controls were from the urban areas. Nearly around half of the study participants, 208 (48.9%), were found in the 25-29 years age group. The mean age of study participants was 26.11 ± 4.05 for cases and 27.57 ± 5.27years for controls. Almost all 135 (95.1%) cases and 272 (95.8%) controls were married. The husbands of the majority of 126 (88.7%) cases and 202 (71.1%) controls were unemployed.

### 3.2. Dietary Habits and Nutritional-Related Characteristics

**Figure 2:**
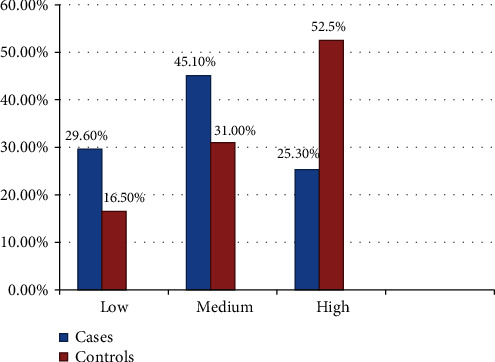
Dietary diversity score of pregnant women attending antenatal care follow-ups in public hospitals in Central Ethiopia, 2019.

Only 51 (35.9%) cases and 31 (10.9%) controls were having meal frequency of ≤ twice per day. Nearly two-thirds of 97 (68.3%) cases and one-third of 94 (33.1%) controls consumed coffee/tea immediately after meals. Nearly one-third of 46 (32.6) cases and 23 (8.1%) controls had a history of forbidden food (food taboo) during pregnancy. Two-thirds of 96 (67.6%) cases and 106 (37.3%) controls did not take additional food/meals during pregnancy (Table 1). Forty-six (29.6%) cases and 47 (16.5%) controls had a low DDS, whereas 64 (45.1%) cases and 88 (31%) controls had medium DDS (Figure 2). Only 31 (21.8%) cases and near to quarter of 81 (28.5%) controls ate dark green leafy vegetables (DGLV) 1-2 times per week. Forty-nine (34.5%) cases and 22 (7.7%) controls did not eat fruit at all. Around two-thirds of 93 (65. 5%) cases and 94 (33.1%) controls had MUAC less than 23 centimeters (Table 2).

## 4. Nutritional-Related Predictors of Anemia among Pregnant Women

Bivariate logistic regression was performed for all independent variables. Multivariate analysis was done for those variables with a *p* value less than 0.25 in the bivariate logistic regression. After adjusting for covariates in multivariable logistic regressions, the odds of having anemia was twice greater among pregnant mothers who had a history of the presence of forbidden food than those who had no history of the presence of forbidden food during their current pregnancy (AOR = 2.02, 95% CI: 1.06-3.86). Anemia was 2.35 times more common among pregnant women who drank tea or coffee immediately after food than their counterparts (AOR = 2.35, 95% CI: 1.39-3.99). Women who had mid-upper arm circumference less than twenty-three were 3.83 times more likely to be anemic than their counterparts (AOR = 3.83, 95% CI: 2.26-6.49).

The current study also revealed that pregnant mothers who did not take additional meals during their current pregnancy were almost two times more likely to be anemic than those who did (AOR = 1.99, 95% CI: 1.17-3.40). Pregnant mothers with MUAC of <23 centimeters were 3.83 times more likely to develop anemia than those who had MUAC of ≥23 centimeters (AOR = 3.83, 95% CI: 2.26-6.49). Similarly, those women who did not consume fruits were four times more likely to be anemic than those who took fruits for at least 1-2 times per week (AOR = 4.05, 95% CI: 1.3-15.47). Women who had low DDS were 3.29 times more likely to develop anemia than those who had high DDS (AOR = 3.29, 95% CI: 1.83-5.90). The odds of being anemic were 2.88 times higher among pregnant women who had medium DDS than those who had high DDS (AOR = 2.88, 95% CI: 1.46-5.70) (Table 3).

## 5. Discussion

The study revealed that the presence of food taboo, taking tea/coffee immediately after food, not taking additional food, mid-upper arm circumference of women, unable to eat fruits, and dietary diversity were identified as nutritional-related factor determinants of anemia among pregnant women attending antenatal care at public hospitals in Central Ethiopia.

Taking tea or coffee immediately after food was positively associated with the occurrence of anemia among pregnant women in this study. This finding is consistent with a study done in Durame Town and West Arsi Zones, Ethiopia, which indicated that taking tea or coffee immediately after food was among the most significant risk factors for the development of anemia during pregnancy [28, 29]. This could be due to drinking tea or coffee without staying at least for 1-2 hours after the food is eaten which inhibits iron absorption which in turn leads to anemia [25].

Not taking additional food was also found to be a predictor of anemia among pregnant women. This finding is similar with a study conducted in Durame and Mekele Towns, where not taking an additional meal during pregnancy was positively associated with the occurrence of anemia [11, 28]. This could be due to the fact that during pregnancy, women require additional nutrients for themselves and growing fetus that can be maintained by increasing frequency of meal.

Furthermore, having mid-upper arm circumference less than 23 centimeters during pregnancy was positively associated with the occurrence of anemia. This finding is in agreement with the studies conducted in Southern Ethiopia and Gode Town and Jijiga City in Eastern Ethiopia [30, 31]. This might be due to malnourished women who are more likely to have micronutrient deficiency that leads to iron deficiency which in turn causes anemia [31, 32].

The risk of developing anemia is higher among pregnant women who did not take fruits than those women who took fruits at least 1-2 times per week. It is comparable with studies conducted in Asosa and West Arsi Zones where pregnant women who did not take fruits were more likely to be anemic as compared to their counterparts [29, 33]. This is because fruits are the source of vitamin C which facilitates iron absorption, and its lack leads to anemia [1].

Another interesting finding of this study but uncommon to other studies in Ethiopia was food taboo or the presence of culture that restricts some food items like butter, fruits, and vegetables during pregnancy due to fear of fetal overdevelopment and attachment of food to the fetus. The presence of food taboo during pregnancy was statistically significantly associated with the occurrence of anemia among pregnant women. This is because unability to consume vegetables like that of DGLV leads to deficiency of nonhem iron that causes iron deficiency anemia. Vitamins A and C enhance iron absorption [1], and intake of vegetarian diet was protective for anemia [16, 34]. In addition, vitamin A is used for the synthesis of RBC and immune function where its lacks lead to lack of hemoglobin and cause an infection that in turn leads to anemia. Moreover, unability to take fruit causes a lack of vitamin C which enhances iron absorption where its deficiency leads to anemia. Restricting pregnant women from taking food leads not only to anemia but also to other macronutrient deficiencies.

The women dietary diversity score was positively associated with the occurrence of anemia in pregnancy in this study. This finding is consistent with the study conducted in Southern Ethiopia with women having low DDS and Mekele Town [11, 35]. The reason for this might be due to the fact that pregnancy is a nutritionally demanding period in women's life. Consequently, pregnant women are compulsory to eat diversified diets than usual to mitigate the problems of micronutrient deficiency [11, 36]. Moreover, increasing individuals' dietary diversity score is interrelated to increasing nutritional adequacy and can be used as a proxy indicator of measuring nutritional adequacy [37].

### 5.1. Limitation of the Study

This study may be susceptible to recall and social desirability biases regarding dietary intake.

## 6. Conclusion

In this study, taking tea or coffee immediately after food, the presence of forbidden food during their current pregnancy, not taking additional food during pregnancy, low dietary diversity score, medium dietary diversity score, MUAC of mother less than twenty-three, and failure to take fruits were identified predictors of anemia among pregnant women.

Therefore, interventions targeted at prevention of anemia among pregnant mothers should emphatically consider the identified predictors. This finding also highlights the need for strong nutritional counseling, dietary advice, and therapy to prevent anemia among pregnant mothers during antenatal care follow-ups along with provision of iron folate supplementation and provision of health education to reverse food taboos. We also recommend further community-based study with a strong study design to determine other determinants of anemia among pregnant women to address those pregnant women who did not attend ANC.

## Figures and Tables

**Table 1:** Dietary habit and nutritional-related characteristics of pregnant women attending ANC care at public hospitals in Central Ethiopia, 2019.

Variables	Cases *n* = 142 (%)	Controls *n* = 284 (%)
*Staple food*		
Injera of teff and wot	98 (68%)	228 (80.3%)
Maize and sorghum	36 (25.4%)	42 (14.8%)
Wheat and barley	8 (5.6%)	14 (4.9%)
*Meal frequency*		
Twice	51 (35.9%)	31 (10.9%)
Three times	74 (52.1%)	136 (47.9%)
Four times and above	17 (12%)	117 (41.2%)
*Presence of forbidden food for pregnant women*		
Yes	46 (32.6)	23 (8.1%)
No	96 (67.4%)	261 (91.9%)
*Status of appetite*		
Decreased	89 (62.7%)	72 (25.4%)
Increased	14 (9.9%)	81 (28.5%)
No change	39 (27.5%)	131 (46.1%)
*Taking tea/coffee immediately after food*		
Yes	97 (68.3%)	94 (33.1%)
No	45 (31.7%)	190 (66.9%)
*Pica*		
Yes	39 (27.5%)	36 (12.7%)
No	103 (72.5%)	248 (87.3%)
*Food aversion*		
Yes	63 (44.4%)	48 (16.9%)
No	79 (55.6%)	236 (83.1)
*Taking additional food*		
Yes	36 (32.4%)	178 (62.7%)
No	96 (67.6%)	106 (37.3%)
*MUAC of mother*		
<23	105 (73.9%)	38 (13.4%)
≥23	37 (26.1%)	246 (86.6%)

**Table 2:** Food frequency of pregnant women attending ANC care at public hospitals in Central Ethiopia, February to April 2019.

Variables	Cases *n* = 142 (%)	Controls *n* = 284 (%)
*Frequency of red meat consumption*		
1-2 times in a week	9 (6.3%)	45 (15.8%)
Once in two weeks	50 (35.2%)	143 (50.4%)
Do not take	78 (55%)	83 (29.2%)
Others^a^	5 (3.5%)	13 (4.6%)
*Frequency of organ meat like liver and kidney*		
Once in two weeks	15 (10.6%)	174 (26.1%)
Do not take	121 (85.2%)	189 (66.5%)
Others^b^	6 (4.2%)	21 (7.4%)
*Frequency of DGLV*		
Daily	13 (9.2%)	45 (15.8%)
Every other day	28 (19.7%)	59 (20.8%)
1-2 times per week	31 (21.8%)	81 (28.5%)
Once in two weeks	40 (28.2%)	72 (25.4%)
Do not take	30 (21.1%)	21 (9.5%)
*Frequency of fruits (mango, avocado, papaya, lemon, orange, etc.)*		
Daily	6 (4.2%)	19 (6.7%)
Every other day	13 (9.2%)	45 (15.8%)
1-2 times per week	29 (20.4%)	85 (29.9%)
Once in two weeks	45 (31.7%)	113 (39.8%)
Do not take	49 (34.5%)	22 (7.7%)
*Frequency of legumes (peas, beans, lentils, soybeans, etc.)*		
Daily	66 (46.5%)	130 (45.8%)
Every other day	28 (19.7%)	45 (15.8%)
1-2 times per week	23 (16.2%)	58 (20.4%)
Once in two weeks	19 (13.4%)	42 (14.8%)
Do not take	6 (4.2%)	9 (3.2%)
*Frequency of egg consumption*		
Daily	8 (5.6%)	21 (7.4%)
Every other day	9 (6.3%)	39 (13.7%)
1-2 times per week	36 (25.4%)	81 (28.5%)
Once in two weeks	55 (38.7%)	115 (40.5%)
Do not take	34 (23.9%)	28 (9.9%)
*Frequency of chicken*		
1-2 times per week	4 (2.8%)	8 (2.8%)
Once in two weeks	22 (15.5%)	71 (25%)
Do not take	116 (81.7%)	205 (72.2%)
*Food made from teff consumption*		
Daily	99 (69.7%)	221 (77.8%)
Every other day	21 (14.8%)	39 (13.7%)
1-2 times per week	17 (12%)	20 (7%)
Once in two weeks	5 (3.5%)	4 (1.4%)
*Frequency of milk and milk product consumption*		
Daily	18 (12.7%)	67 (23.6%)
Every other day	31 (21.8%)	74 (26.1%)
1-2 times per week	40 (28.2%)	87 (30.6%)
Once in two weeks	31 (21.8%)	39 (13.7%)
Do not take	22 (15.5%)	17 (6%)

^a^Daily and every other day. ^b^1-2 times per week and every other day.

**Table 3:** Nutritional-related predictors of anemia among pregnant women attending antenatal care follow-ups in public hospitals in Central Ethiopia, April 2019 (multivariable analysis).

Variables	Cases *n* = 142 (%)	Controls *n* = 284 (%)	COR (95% CI)	AOR (95% CI)
*Taking tea/coffee after food*				
Yes	97 (68.3%)	94 (33.1%)	1	1
No	45 (31.7%)	190 (66.9%)	4.36 (2.83-6.71)	2.35 (1.39-3.99)^∗∗^
*Presence of forbidden food during pregnancy (food taboo)*				
Yes	46 (32.6)	23 (8.1%)	1	1
No	96 (67.4%)	261 (91.9%)	2.76 (1.71-6.46)	2.02 (1.06-3.86)^∗^
*Mid-upper arm circumference of mother in centimeters*				
<23	105 (73.9%)	38 (13.4%)	5.79 (3.71-9.04)	3.83 (2.26-6.49)^∗∗^
≥23	37 (26.1%)	246 (86.6%)	1	1
*Taking an additional food/meal during pregnancy*				
Yes	36 (32.4%)	178 (62.7%)	1	1
No	96 (67.6%)	106 (37.3%)	3.51 (2.29-5.37)	1.99 (1.17-3.40)^∗∗^
*Frequency of taking (eating) fruits during pregnancy*				
Daily	6 (4.2%)	19 (6.7%)	1	1
Every other day	13 (9.2%)	45 (15.8%)	0.91 (0.3-2.77)	1.34 (0.36-4.99)
1-2 times per week	29 (20.4%)	85 (29.9%)	1.08 (0.39-2.97)	1.18 (0.35-3.94)
Once in 2 weeks	45 (31.7%)	113 (39.8%)	1.26 (0.47-3.36)	1.48 (0.46-4.82)
Do not take	49 (34.5%)	22 (7.7%)	7.05 (2.48-20.09)	4.05 (1.3-15.47)^∗^
*Dietary diversity score of the women*				
Low	42 (29.6%)	47 (16.5%)	3.01 (1.85-4.89)	3.29 (1.83-5.9)^∗∗^
Medium	64 (45.1%)	88 (31%)	3.7 (1.13-6.43)	2.88 (1.46-5.7)^∗∗^
High	36 (25.3%)	149 (52.5%)	1	1

AOR: adjusted odds ratio; COR: crude odds ratio; CI: confidence interval; 1: reference group. ^∗^Statistically significant at *p* value < 0.05. ^∗∗^Statistically significant at *p* value < 0.01.

## Data Availability

The data used to support the findings of this study are available from the corresponding author upon request.

## Abbreviations

ANC: Antenatal care
DDS: Dietary diversity score
DGLV: Dark green leafy vegetables
IDA: Iron deficiency anemia
ID: Iron deficiency
MUAC: Mid-upper arm circumference
WDDS: Women dietary diversity score
WHO: World Health Organization.

## References

[1] Benoist B., McLean E., Egll I., Cogswell M. (2008). *Worldwide prevalence of anaemia 1993-2005: WHO global database on anaemia*.

[2] Karaoglu L., Pehlivan E., Egri M. (2010). The prevalence of nutritional anemia in pregnancy in an east Anatolian Province, Turkey. *BMC Public Health*.

[3] Stevens G. A., Finucane M. M., de-Regil L. M. (2013). Global, regional, and national trends in haemoglobin concentration and prevalence of total and severe anaemia in children and pregnant and non-pregnant women for 1995–2011: a systematic analysis of population-representative data. *The Lancet Global Health*.

[4] World Health Organization (2015). The global prevalence of anaemia in 2011.

[5] Central Statistical Agency CSAE (2017). *Ethiopia Demographic and Health Survey 2016*.

[6] Balcı Y. I., Karabulut A., Gürses D., Çövüt İ. E. (2012). Prevalence and risk factors of anemia among adolescents in Denizli, Turkey. *Iranian Journal of Pediatrics*.

[7] Umeta M., Haidar J., Demissie T., Akalu G., Ayana G. (2008). Iron deficiency anaemia among women of reproductive age in nine administrative regions of Ethiopia. *Ethiopian Journal of Health Development*.

[8] World Health Organization (2014). *Global nutrition targets 2025: Policy Brief Series*.

[9] Kennedy E., Fekadu H., Ghosh S. (2016). Implementing multisector nutrition programs in Ethiopia and Nepal: challenges and opportunities from a stakeholder perspective. *Food and Nutrition Bulletin*.

[10] Ethiopia GotFDRo National nutrition programme implementing sectors declaration.

[11] Abriha A., Yesuf M. E., Wassie M. M. (2014). Prevalence and associated factors of anemia among pregnant women of Mekelle town: a cross sectional study. *BMC Research Notes*.

[12] Alem M., Enawgaw B., Gelaw A., Kenaw T., Seid M., Olkeba Y. (2013). Prevalence of anemia and associated risk factors among pregnant women attending antenatal care in Azezo Health Center Gondar Town, Northwest Ethiopia. *Journal of Interdisciplinary Histopathology*.

[13] Gebreweld A., Tsegaye A. (2018). Prevalence and factors associated with anemia among pregnant women attending antenatal clinic at St. Paul’s Hospital Millennium Medical College, Addis Ababa, Ethiopia. *Advances in Hematology*.

[14] Gebreslasie K. (2016). Preterm birth and associated factors among mothers who gave birth in Gondar Town health institutions. *Advances in Nursing*.

[15] Asrie F. (2017). Prevalence of anemia and its associated factors among pregnant women receiving antenatal care at Aymiba Health Center, Northwest Ethiopia. *Journal of Blood Medicine*.

[16] Melku M., Addis Z., Alem M., Enawgaw B. (2014). Prevalence and predictors of maternal anemia during pregnancy in Gondar, Northwest Ethiopia: an institutional based cross-sectional study. *Anemia*.

[17] Gedefaw L., Ayele A., Asres Y., Mossie A. (2015). Anaemia and associated factors among pregnant women attending antenatal care clinic in Walayita Sodo Town, Southern Ethiopia. *Ethiopian Journal of Health Sciences*.

[18] Lelissa D., Yilma M., Shewalem W. (2015). Prevalence of anemia among women receiving antenatal care at Boditii Health Center, Southern Ethiopia. *Age*.

[19] Shitie D., Zewde T., Molla Y. (2018). Anemia and other hematological profiles of pregnant women attending antenatal care in Debre Berhan Referral Hospital, North Shoa, Ethiopia. *BMC Research Notes*.

[20] Ayano B., Amentie B. (2018). Assessment of prevalence and risk factors for anemia among pregnant mothers attending ANC clinic at Adama Hospital Medical Collage, Adama, Ethiopia, 2017. *Journal of Gynecology and Obstetrics*.

[21] Gebre A., Mulugeta A. (2015). Prevalence of anemia and associated factors among pregnant women in north western zone of Tigray, northern Ethiopia: a cross-sectional study. *Journal of Nutrition and Metabolism*.

[22] Mekonnen F. A., Ambaw Y. A., Neri G. T. (2018). Socio-economic determinants of anemia in pregnancy in North Shoa Zone, Ethiopia. *PLoS One*.

[23] Office WSzH (2019). *Health managment information system third quarterly report*.

[24] Kennedy G., Ballard T., Dop M. C. (2011). *Guidelines for Measuring Household and Individual Dietary Diversity*.

[25] World Health Organization (2017). *Nutritional Anaemias: Tools for Effective Prevention and Control*.

[26] Tadesse S. E., Seid O., G/Mariam Y. (2017). Determinants of anemia among pregnant mothers attending antenatal care in Dessie town health facilities, northern central Ethiopia, unmatched case -control study. *PLoS One*.

[27] Sperandei S. (2014). Understanding logistic regression analysis. *Biochemia Medica*.

[28] Weldekidan F., Kote M., Girma M., Boti N., Gultie T. (2018). Determinants of anemia among pregnant women attending antenatal clinic in public health facilities at Durame Town: unmatched case control study. *Anemia*.

[29] Obse N., Mossie A., Gobena T. (2013). Magnitude of anemia and associated risk factors among pregnant women attending antenatal care in Shalla Woreda, West Arsi Zone, Oromia Region, Ethiopia. *Ethiopian Journal of Health Sciences*.

[30] Addis Alene K., Mohamed Dohe A. (2014). Prevalence of anemia and associated factors among pregnant women in an urban area of eastern Ethiopia. *Anemia*.

[31] Bereka S., Gudeta A., Reta M., Ayana L. (2017). Prevalence and associated risk factors of anemia among pregnant women in rural part of Jijiga City, Eastern Ethiopia: a cross sectional study. *Journal of Pregnancy and Child Health*.

[32] Derso T., Abera Z., Tariku A. (2017). Magnitude and associated factors of anemia among pregnant women in Dera District: a cross-sectional study in Northwest Ethiopia. *BMC Research Notes*.

[33] Abay A., Yalew H. W., Tariku A., Gebeye E. (2017). Determinants of prenatal anemia in Ethiopia. *Archives of Public Health*.

[34] Viveki R., Halappanavar A., Viveki P., Halki S., Maled V., Deshpande P. (2012). Prevalence of anaemia and its epidemiological determinants in pregnant women. *Al Ameen Journal of Medical Sciences*.

[35] Lebso M., Anato A., Loha E. (2017). Prevalence of anemia and associated factors among pregnant women in Southern Ethiopia: a community based cross-sectional study. *PLoS One*.

[36] Okube O. T., Mirie W., Odhiambo E., Sabina W., Habtu M. (2016). Prevalence and factors associated with anaemia among pregnant women attending antenatal clinic in the second and third trimesters at Pumwani Maternity Hospital, Kenya. *Open Journal of Obstetrics and Gynecology*.

[37] Wen L. M., Flood V. M., Simpson J. M., Rissel C., Baur L. A. (2010). Dietary behaviours during pregnancy: findings from first-time mothers in Southwest Sydney, Australia. *International Journal of Behavioral Nutrition and Physical Activity*.

